# Remittent and Typhoid Fevers

**Published:** 1847-02

**Authors:** 


*Remittent and Typhoid Fevers.*—In our last number, we published a letter from Prof. Bartlett, requesting information respecting the present and past prevalence of Typhoid fever in this section of country, and its relative frequency, now and heretofore, compared with Remittent fever. We read the letter at the last meeting of the Medical association of this city, and the following is a succinct account of the substance of the remarks to which it gave rise. We quote from the records of the meeting by the Secretary, Dr. Geo. N. Burwell:

"Dr. F. read a letter from Prof. Bartlett on the subject of Fever; requesting information respecting the forms of fever most prevalent in this state. Dr. J. Trowbridge remarked that the malarial fevers so prevalent twenty years ago in this section of the country had disappeared to a great extent, and were replaced by the Typhoid forms of fever. The Typhoid fever was not prevalent when bilious fevers prevailed, although some cases of the latter partook of the typhoid form when occuring late in the fall.

Dr. Sprague mentioned an epidemic of Typhus fever which occurred at Springville (Erie Co,) in the winter of 1826 and 27. It was evidently contagious, and extended through a circuit of fifty miles. Some of the patients were afflicted, during the course of the fever, with numerous furunculi. In that district which is very high and hilly, no malarial fever ever occurs.

Dr. B. Burwell remarked that Ague and Fever had been comparatively rare since the last year (1834) of cholera. That epidemic changed the character of bilious diseases.

Dr. Loomis had remarked the same change after cholera, in Wayne Co, this state, where he then practiced.

All agreed upon the great severity of Bilious diseases through western New-York and the Genesee Valley in the summer of 1828, when this district was as sickly as any portion of the western states, during the past summer."

Drs. Trowbridge, B. Burwell, and Sprague, have practised in this locality for a great number of years, and have had ample opportunities for observation with respect to its diseases. We would add to their longer and more abundant experience, that the fact of a change in the character of febrile diseases has been apparent to us since we commenced the practice of medicine here in 1836. Coming from a district of New England in which the fever called Typhoid prevailed almost exclusively, we found the Remittent, at that time, equally predominant here. We do not recollect meeting with a single case of Typhoid fever during the first few years of our residence in this city, although we are not prepared to say cases of that form of fever may not have occurred occasionally, under our own observation, without being accurately diagnosticated. The distinctive characters appertaining to the two forms of febrile disease had not been so fully delineated, or rather had not become so familiar to the profession as now. At all events we are free to say that we were not so prepared to appreciate them as of late years. Our attention to the existence of typhoid fever in this region was first excited by its occurrence in an epidemic form at a small settlement, called North Boston, about 18 miles from this city. This was in 1843.

A report of this epidemic was communicated by us for the Am. Jour. of Med, Sciences, July. 1845. The fact that the characters appertaining to this epidemic were so different from those of the fever commonly met with in this place and its vicinity, was adduced as a strong point in favor of the position that it was imported and propagated by contagion. Although we see no reasons to recede from this position, we are ready to admit that the point referred to is weakened somewhat by the frequent occurrence of undoubted Typhoid fever, since that epidemic, both in this city and neighborhood, under circumstances which render the supposition of contagion gratuitous. We should say that the greater proportion of cases of fever since 1843, which have fallen under our immediate notice, have been distinctly Typhoid. Several of these have been fine exemplifications of the fever so admirably described by Louis. This has been the experience, also, of those of our brethren with whom we have been in the habit of conversing on the subject, both in the city and its vicinity, for the past two or three years. That this form of fever has to a great extent superseded the Remittent form, has been a matter of frequent remark for some time, and in a brief enumeration of the distinctive traits of Remittent, Typhus and Typhoid fever, published by us in the first volume of thls Journal, we mentioned this as a sentiment generally entertained by the Profession in this region. That some allowance is to be made from the fact, already referred to, that the boundary lines between the two forms are now much more clearly drawn, and have been rendered more familiar to practitioners, we regard as highly probable; yet, we think, there cannot be a doubt that a striking change has taken place within a few years past, and that Typhoid fever, from having been, to say the least, of unfrequent occurrence, has become frequent, and is becoming more and more so, Remittents diminishing in frequency after the same ratio.

